# 局部放疗联合化疗在广泛期小细胞肺癌患者中的作用

**DOI:** 10.3779/j.issn.1009-3419.2015.05.04

**Published:** 2015-05-20

**Authors:** 頔 吴, 健 方, 鋆 聂, 玲 戴, 筱玲 陈, 洁 张, 维亨 胡, 金娣 韩, 向娟 马, 广明 田, 森 韩, 皆然 龙, 洋 王

**Affiliations:** 100142 北京，北京大学肿瘤医院暨北京市肿瘤防治研究所，恶性肿瘤发病机制及转化研究教育部重点实验室，胸部肿瘤内二病房 Key Laboratory of Carcinogenesis and Translational Research (Ministry of Education), the Second Department of Chest Cancer, Peking University Cancer Hospital and Institute, Beijing 100142, China

**Keywords:** 肺肿瘤, 放疗, 全脑预防性照射治疗, 局部治疗, 生存期, Lung neoplasms, Radiotherapy, Prophylactic cranial irradiation, Local treatment, Survival time

## Abstract

**背景与目的:**

化疗作为广泛期小细胞肺癌（extensive-stage small cell lung cancer, ES-SCLC）的主要治疗方法，其有效率高，但容易发生快速耐药。局部治疗作为姑息治疗方法，能够缓解局部症状和提高患者生活质量，但是能否延长生存期尚缺乏证据。本研究旨在分析在全身化疗基础上的局部放疗对ES-SCLC患者生存的作用与影响。

**方法:**

回顾性分析302例ES-SCLC患者临床资料，采用*Kaplan*-*Meier*法及*Cox*多因素回归分析预后因素。

**结果:**

全组患者中位无进展生存期（progression-free survival, PFS）4.4个月，中位生存期（median survival time, MST）10.4个月，1年生存率37.8%；2年生存率10.2%；3年生存率4.4%。原发灶放疗+化疗组和单纯化疗组患者MST分别为14.3个月和8.2个月（*P* < 0.01）；多处转移灶局部治疗、一处转移灶局部治疗和未行转移灶局部治疗患者MST分别为18.7个月、12.3个月和8.9个月（*P* < 0.01）；主动性的转移灶局部治疗、被动性的转移灶局部治疗和未行转移灶局部治疗患者MST分别为16.0个月、10.9个月和9.4个月（*P* < 0.01）；全脑预防性照射治疗（prophylactic cranial irradiation, PCI）和未行PCI患者MST分别为19.8个月和9.9个月（*P* < 0.01）。原发灶放疗、转移灶局部治疗和PCI是ES-SCLC患者的独立预后因素（*P* < 0.01）。

**结论:**

原发灶放疗、转移灶局部治疗和PCI能够延长ES-SCLC患者生存期。

## Abstract (en)

**Background and objective:**

Chemotherapy is a highly efficient primary treatment for extensive-stage small cell lung cancer (ES-SCLC). However, patients receiving such treatment are prone to develop drug resistance. Local treatment is palliative and thus can alleviate the local symptoms and improve quality of life, but limited evidence is available for prolonging survival. Hence, this study evaluated the role of local treatment in chemotherapy of patients with ES-SCLC.

**Methods:**

A total of 302 ES-SCLC cases were enrolled in this retrospective study. Prognostic factors were analyzed by *Kaplan*-*Meier* and *Cox* multivariate proportional hazards model.

**Results:**

Median progression-free survival (PFS) and median survival time (MST) of the patients were 4.4 and 10.4 months, respectively. 1-, 2-, and 3-year survival rates were 37.8%, 10.2% and 4.4%, correspondingly. The MST of the primary tumor radiotherapy plus chemotherapy group was 14.3 months, whereas that of the chemotherapy group was 8.2 months (*P* < 0.01). The MSTs of multiple-site, single-site, and non-metastasis local treatments were 18.7, 12.3 and 8.9 months, respectively (*P* < 0.01). The MSTs of initiative, passive, and non-metastasis local treatments were 16.0, 10.9 and 9.4 months, correspondingly (*P* < 0.01). The MSTs of patients with prophylactic cranial irradiation (PCI) and those without PCI were 19.8 and 9.9 months, respectively (*P* < 0.01). Primary tumor radiotherapy, metastasis local treatment, and PCI were independent prognostic factors for ES-SCLC.

**Conclusion:**

Primary tumor radiotherapy, metastasis local treatment, and PCI can significantly improve survival in patients with ES-SCLC.

小细胞肺癌（small cell lung cancer, SCLC）患者占全部肺癌患者的15%左右^[1]^。因其倍增时间短，肿瘤细胞增殖快，易发生早期转移，约60%的SCLC患者在初治时诊断为广泛期（extensive-stage, ES）。目前对于广泛期小细胞肺癌（extensive-stage small cell lung cancer, ES-SCLC）患者，治疗仍以化疗为主，化疗有效率高，能使大多数患者迅速缓解症状，但容易发生快速耐药。近40年来，ES-SCLC患者的中位生存期（median survival time, MST）仍在9个月-11个月^[2]^。原发灶及转移灶局部治疗作为姑息治疗方法，能够缓解局部症状并提高患者生活质量，但是上述方法能否延长生存期尚缺乏证据。本研究回顾性分析302例ES-SCLC患者的局部治疗与生存关系，旨在分析局部治疗对于改善ES-SCLC患者生存的作用。

## 1 资料与方法

### 1.1 临床资料

收集2008年8月-2014年5月北京大学肿瘤医院胸部肿瘤内二科病房收治的经组织病理学或细胞学证实为SCLC，采用美国退伍军人医院制定的分期标准，经影像学确诊为ES-SCLC患者的临床资料302例，其中失访27例（8.9%），可分析病例275例。该组患者至少接受1周期以上化疗并有评效结果，排除单纯手术或放疗的患者。中位年龄61岁（34岁-81岁），男性212例、女性63例。

### 1.2 治疗方法

ES-SCLC患者以化疗为主，根据肿瘤局部退缩情况给予原发灶放疗，部分患者给予其他局部治疗，包括局部放疗、肝射频消融。对缓慢增长、无症状的转移灶给予的择期性的局部治疗，本研究定义为主动性的局部治疗；对迅速增长、有局部症状的转移灶给予的限期性的局部治疗，本研究定义为被动性的局部治疗。部分放化疗后缓解患者接受全脑预防性照射治疗（prophylactic cranial irradiation, PCI）。化疗方案包括EP或EC方案（卡铂、顺铂或奈达铂+依托泊苷）219例，CODE（顺铂+长春新碱+阿霉素+依托泊苷）13例，IP（伊立替康+顺铂）18例，Topotecan+DDP（拓扑替康+顺铂）6例，CAV（环磷酰胺+多柔吡星+长春新碱）6例，TAX+CBP（紫杉醇+卡铂）3例，Topotecan（拓扑替康）2例，VP-16（依托泊苷）2例，VCR+DDP（长春新碱+顺铂）2例，TAX+EPI（紫杉醇+表柔比星）1例，GEM+CBP（吉西他滨+卡铂）1例，VCR+VP-16（长春新碱+依托泊苷）1例，DDP+VM26（顺铂+替尼泊苷）1例。

### 1.3 生存期

观察终点为死亡或末次随访，末次随访日期为2014年10月21日，总生存期（overall survival, OS）指开始治疗日期至死亡或末次随访日期，无进展生存期（progression-free survival, PFS）指开始治疗日期至肿瘤进展或死亡日期。以月为单位。

### 1.4 统计学方法

所有数据录入SPSS 19.0软件数据库，计数资料比较采用卡方检验，单因素分析采用*Kaplan*-*Meier*法，生存率曲线组间比较采用*Log*-*rank*检验，选择单因素分析有统计学意义的变量进入多因素*Cox*比例风险回归模型分析。*P* < 0.05认为差异有统计学意义。

## 2 结果

### 2.1 临床特点统计

ES-SCLC患者临床特征包括：性别、年龄、美国东部肿瘤协作组（Eastern Cooperative Oncology Group, ECOG）评分、转移部位、转移特点、局部治疗情况及一线化疗疗效，详见表 1。

**1:** ES-SCLC患者临床特征 Clinical characteristics of ES-SCLC patients

Clinical characteristics	*n*	Percentage (%)
Total	275	100
Gender		
< Male	212	77
< Female	63	23
Age (yr)		
< 65	176	64
≥65	99	36
ECOG		
0-1	237	86
< 2-3	38	14
Brain metastases		
< Yes	60	22
< No	215	78
Liver metastases		
< Yes	74	27
< No	201	73
Bone metastases		
< Yes	76	28
< No	199	72
Adrenal metastases		
< Yes	46	17
< No	229	83
Metastases characteristics		
< Intra-thoracic metastases	37	13
< Single site distant metastases	61	22
< Muti-site distant metastases	177	65
Primary tumor radiotherapy		
< Yes	122	44
< No	153	56
PCI		
< Yes	34	12
< No	241	88
Local treatment of metastases		
< Yes	109	40
< No	166	60
Efficacy of first-line treatment		
< CR	13	5
< PR	187	68
< SD	36	13
< PD	39	14
EP: etoposide+cisplatin; EC: etoposide+carboplatin; CR: complete remission; PR: partial remission; SD: stable disease; PD: progressive disease; EOCG: Eastern Cooperative Oncology Group; ES-SCLC: extensive-stage small cell lung cancer.		

### 2.2 随访结果

全组患者中位PFS 4.4个月，MST 10.4个月，客观缓解率73%。随访时间截止到2014年10月21日，随访时间为4.7个月-74.3个月，中位随访时间30.7个月。到随访截止日期，死亡患者246例（89.5%），1年生存率37.8%；2年生存率10.2%；3年生存率4.4%。

### 2.3 原发灶放疗

根据ES-SCLC患者是否接受原发灶放疗，将患者分为放化疗组（化疗+原发灶放疗）和化疗组（单纯化疗）。其中放化疗组患者122例，化疗组患者153例。MST分别为14.3个月和8.2个月。两组临床特征比较可见放化疗组高龄患者比例更高，见表 2。放化疗组OS优于化疗组（*P* < 0.01，图 1）。

**2:** ES-SCLC患者局部治疗各组间临床特征比较 Clinical characteristics of ES-SCLC patients between local treatment groups

Clinical characteristics	*n*	Primary tumor radiotherapy plus chemotherapy/Chemotherapy	None/Single-site/Mutiple-site local treatment	None/Passive/Initiative treatment	PCI/No PCI
Total	275	122/153	166/85/24	166/43/66	34/241
Gender					29/183
Male	212	90/122	131/62/19	131/28/53	5/58
Female	63	32/31	35/23/5	35/15/13	
Age (yr)					23/153
< 65	176	72/104*	107/53/16	107/24/45	11/88
≥65	99	50/49	59/32/8	59/19/21	
ECOG					33/204
0-1	237	108/129	143/75/19	143/34/60	1/37
2-3	38	14/24	23/10/5	23/9/6	
Brain metastases					——
Yes	60	27/33	19/29/12*	19/18/23*	——
No	215	95/120	147/56/12	147/25/43	
Chemotherapy scheme					31/188
EP or EC	219	97/122	134/71/14	134/35/50	3/53
Not EP or EC	56	25/31	32/14/10	32/8/16	
^*^: *P* < 0.05.					

**1:**
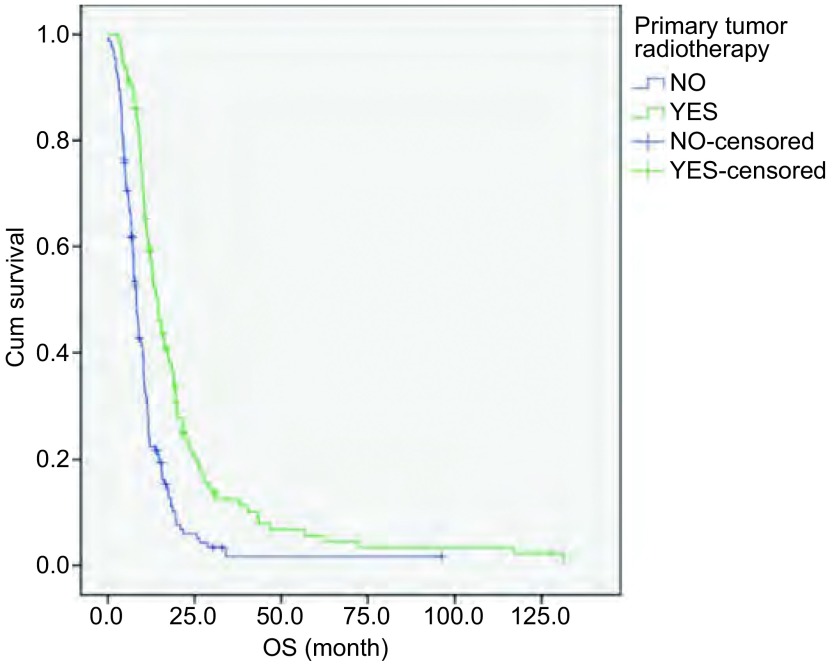
放化疗组与化疗组ES-SCLC患者生存曲线（*Log*-*rank*检验，*P* < 0.01） Survival curves of ES-SCLC patients with primary tumor radiotherapy plus chemotherapy (YES) and chemotherapy (NO) (*Log*-*rank* test, *P* < 0.01)

### 2.4 转移灶局部治疗

转移灶局部治疗方式及例数见表 3。在275例ES-SCLC患者中，行多部位转移灶局部治疗患者24例，单部位转移灶局部治疗患者85例，未行转移灶局部治疗患者166例。MST分别为18.7个月、12.3个月和8.9个月。三组临床特征比较可见未行转移灶局部治疗组中无脑转移患者比例更高（表 2）。三者比较OS差异有统计学意义（*P* < 0.01，图 2A）。

**3:** 转移灶局部治疗情况 Local treatment of metastases

Treatment	*n*
WBRT	63
Bone radiotherapy	21
Cervical lymph nodes radiotherapy	12
Adrenal radiotherapy	3
Pancreas radiotherapy	3
Liver radiotherapy	2
Other lymph nodes radiotherapy	5
Gamma knife	16
Spinal radiotherapy	1
Liver radiofrequency	3
WBRT: whole brain radiotherapy; Cervical lymph nodes include neck, supraclavicular lymph nodes, infraclavicular lymph nodes and submaxillary lymph nodes; Other lymph nodes include celiac lymph nodes, inguinal lymph nodes and axillary lymph nodes.	

**2:**
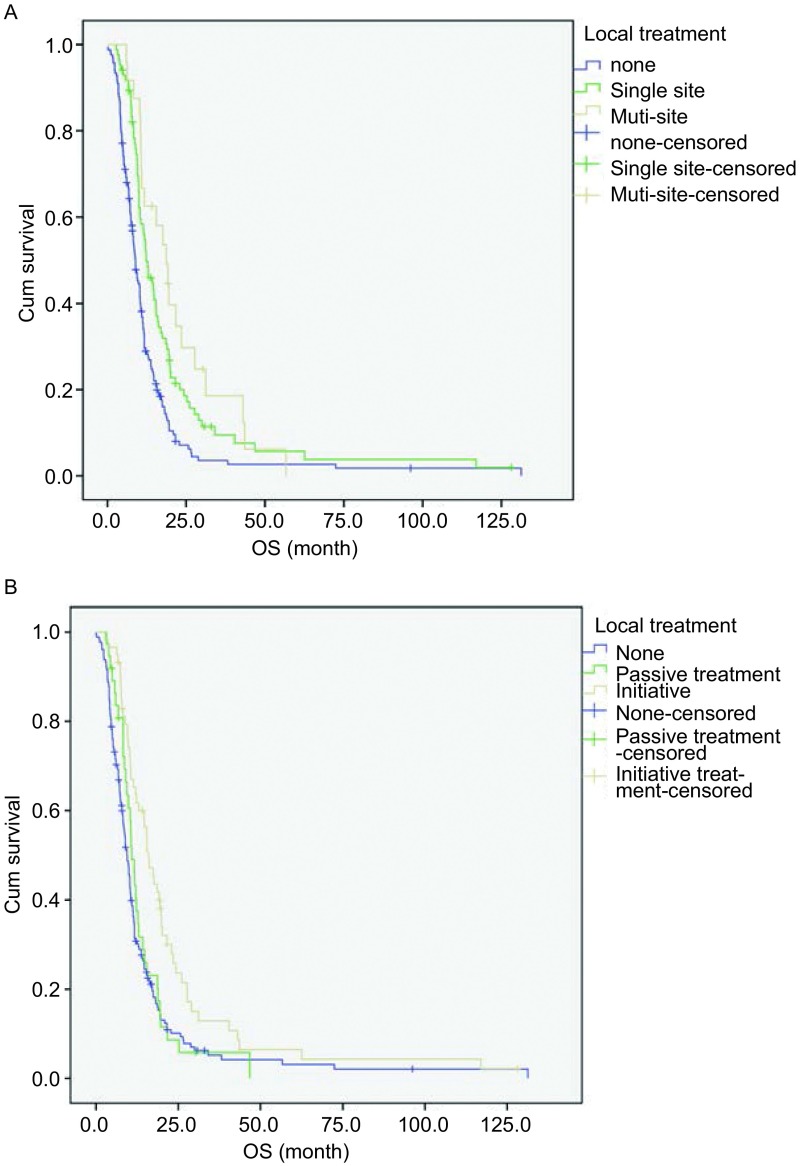
转移灶局部治疗生存曲线（*Log*-*rank*检验）。A：局部治疗部位：未行局部治疗、单部位与多部位局部治疗（*P* < 0.01）；B：局部治疗时机：未行局部治疗、被动与主动性局部治疗（*P* < 0.01） Survival curves of local treatment of metastases (*Log*-*rank* test). A: Local treatment site: none, single-site and multiple-site local treatment of metastases (*P* < 0.01); B: Local treatment timing: none, passive and initiative local treatment of metastases (*P* < 0.01)

另外，在275例ES-SCLC患者中，66例患者行主动性的局部治疗，43例患者行被动性的局部治疗，另有166例患者未行转移灶局部治疗。MST分别为16.0个月、10.9个月和9.4个月。三组临床特征比较可见未行转移灶局部治疗组中无脑转移患者比例更高（表 2）。三者比较OS差异有统计学意义（*P* < 0.01，图 2B）。

### 2.5 PCI

在275例ES-SCLC患者中，34例患者行PCI，其余241例患者未行PCI，MST分别为19.8个月和9.9个月。两组临床特征无统计学差异（表 2）。两组比较OS差异有统计学意义（*P* < 0.01，图 3）。

**3:**
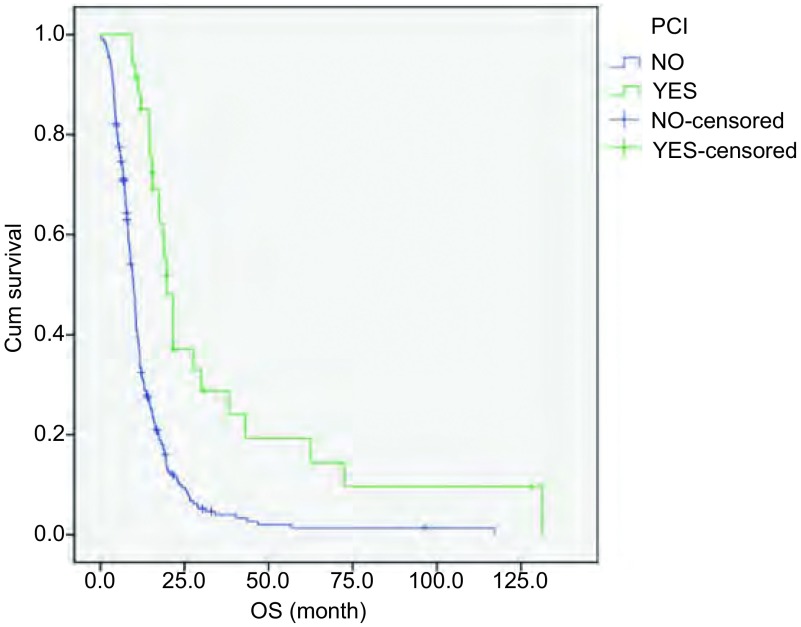
PCI与非PCI ES-SCLC患者生存曲线（*Log*-*rank*检验，*P* < 0.01） Survival curves of ES-SCLC patients with PCI (YES) and no PCI (NO)(*Log*-*rank* test, *P* < 0.01)

### 2.6 单因素分析

单因素分析显示行原发灶放疗、转移灶局部治疗及PCI患者OS延长（*P* < 0.01）。在多部位、单部位和未行转移灶局部治疗组间比较中，前两者之间OS无差异，前两者较未行转移灶局部治疗组OS延长（*P* < 0.01）。在主动性、被动性和未行转移灶局部治疗组间比较中，后两者之间OS无差异，主动性转移灶局部治疗较后两者OS延长（*P* < 0.01）。性别、年龄、治疗前ECOG及一线化疗方案不影响预后（表 4）。

**4:** ES-SCLC生存期的单因素分析 Single analysis for survival of ES-SCLC

Factor	*n*	MST (mo)	Range (mo)	*P*
Total	275	10.4	1.0-43.6	
Gender				0.328
Male	212	10.3	1.0-43.1	
Female	63	11.7	4.1-43.6	
Age (yr)				0.400
< 65	176	10.5	2.3-43.1	
≥65	99	10.4	1.0-43.6	
ECOG before treatment				0.520
0	15	8.3	4.2-21.6	
1	222	10.5	1.0-43.6	
2	33	10.6	2.3-33.0	
3	5	7.2	1.2-27.0	
Thoracic radiotherapy				< 0.001
Yes	122	14.3	3.1-43.6	
No	153	8.2	1.0-34.1	
Prophylactic cranial irradiation				< 0.001
Yes	34	19.8	9.2-43.1	
No	241	9.9	1.0-43.6	
Metastasis local treatment site				< 0.001^*^
Single	85	12.3	2.8-34.1	
Mutiple	24	18.7	6.0-43.6	
None	166	8.9	1.0-28.8	
Metastasis local treatment timing				0.001^**^
Initiative	66	16.0	2.8-43.6	
Passive	43	10.9	4.4-30.3	
None	166	9.4	1.0-28.8	
Chemotherapy scheme				0.854
EP or EC	219	10.9	1.0-43.6	
Not EP or EC	56	10.3	4.1-30.3	
^*^: OS of patients who did not receive local treatment of metastases compared with single-site, multi-site local treatment were statistically different (*P* < 0.01). ^**^: OS of patients who received initiative local treatment of metastases compared with passive, none-local treatment of metastases were statistically different (*P* < 0.01).				

### 2.7 多因素分析

多因素分析显示原发灶放疗、转移灶局部治疗及PCI是独立预后因素（*P* < 0.01）（表 5）。

**5:** ES-SCLC *Cox*回归多因素分析 *Cox* regression of variables in the equation of ES-SCLC

Factor	*P*	RR	95%CI
Primary tumor radiotherapy	0.005	0.647	0.477-0.876
Prophylactic cranial irradiation	< 0.001	0.397	0.253-0.624
Metastasis local treatment site	0.001	0.609	0.453-0.818
Metastasis local treatment timing	0.374	1.116	0.876-1.422

## 3 讨论

目前ES-SCLC的治疗以化疗为主。对于ES-SCLC患者，单纯的化疗能够缓解症状并延长大多数患者的生存时间，但是，长期生存的患者仍然是极少数^[3, 4]^。尽管其对于化疗的客观缓解率能达到60%-80%，但MST也仅有9个月左右^[5]^。本研究中因部分患者加入了局部治疗，全组MST 10.4个月，较文献报道时间略优。

局限期的SCLC采用放化疗作为标准治疗模式。对于ES-SCLC，原发灶放疗通常仅用于缓解局部症状^[6]^。本研究以化疗为基础，根据肿瘤局部退缩情况给予ES-SCLC患者原发灶放疗。在原发灶放疗的患者中，大部分为一线化疗达到PR、CR后进行原发灶巩固性放疗，少数因一线化疗SD，或治疗后PD为加强原发灶局部控制行原发灶放疗。对于老年患者，可能存在较多合并症，以及化疗减量和治疗延期情况，从而间接影响治疗疗效。然而尽管本研究中放化疗组较化疗组高龄患者比例更高，但前者仍显示出显著性生存优势，MST分别为14.3个月和8.2个月（*P* < 0.01），并且在多因素分析中显示，原发灶放疗为保护性预后因素（RR=0.647, *P* < 0.01）。可见原发灶放疗能够显著延长ES-SCLC患者生存期。此结果与既往文献报道相一致。

朱慧等^[7]^的回顾性研究分析了119例ES-SCLC患者，60例进行了原发灶放疗+化疗，59例单纯进行化疗。一线化疗方案采用EP/EC，原发灶放疗总剂量40 Gy-60 Gy，全组MST 13个月，其中原发灶放疗+化疗组与化疗组MST分别为17个月和9.3个月（*P* < 0.01）；在Ou等^[8]^的回顾性研究中，接受原发灶放疗的ES-SCLC患者1, 204例，未行原发灶放疗患者2, 224例，多因素分析显示原发灶放疗为独立保护性因素（HR=0.721; *P* < 0.01）；在一项纳入433例ES-SCLC患者的*meta*分析结果中显示，原发灶放疗+化疗组与化疗组比较，死亡风险降低14%（HR=0.86, *P* < 0.01）^[9]^；Jeremic等^[10]^的一项前瞻性临床研究将化疗3个周期后远处转移病灶CR、原发病灶CR或PR的ES-SCLC患者随机分为同步放化疗组和单纯化疗组，原发灶放疗剂量为45 Gy/30次/3周，两组的MST分别为17个月和11个月；近期一项Ⅲ期随机对照临床研究共入组498例ES-SCLC患者，所有入组患者均行PCI，尽管原发灶放疗+化疗组较化疗组OS无明显差异，但2年生存率明显升高，分别为13%和3%（*P* < 0.01）^[11]^。本研究中放化疗组OS较部分文献报道短，分析可能与化疗后SD、PD患者纳入放化疗组有关。由此推测化疗后达到PR、CR的患者给予原发灶放疗，可能较化疗后达到SD、PD患者更能带来生存获益，然而原发灶放疗的最佳时机尚需要前瞻性研究进行探讨。

转移灶的局部治疗能够缓解ES-SCLC患者的局部症状（例如，疼痛性骨转移灶，脊髓压迫或脑水肿）^[12-14]^，但对于转移灶局部治疗能否延长SCLC患者生存期，却未见文献报道。本研究结果显示：转移灶局部治疗是ES-SCLC的保护性独立预后因素（RR=0.609, *P* < 0.01），转移灶局部治疗能够降低ES-SCLC患者39.1%的死亡风险。在转移灶局部治疗的组间临床特征比较中，未行转移灶局部治疗组的无脑转移患者比例更高，提示未行转移灶局部治疗组患者可能处在更早的疾病病程中，然而其OS却较单部位、多部位转移灶局部治疗组更短。而多处转移的ES-SCLC应具有较差预后，但本研究中多处转移灶局部治疗组却获得了不劣于单部位转移灶局部治疗组的OS，均可说明转移灶的局部治疗可能的确为患者带来了OS的延长。而主动性的局部治疗较被动性的局部治疗患者OS延长，也侧面说明在转移灶迅速增长、出现症状前给予局部控制可能为患者带来更多的获益。而被动性局部治疗组与未行转移灶局部治疗组OS无差异，也说明了当转移灶进入迅速增长期，出现临床症状后再行局部治疗，仅能缓解局部症状，无法带来更多生存的获益。但是由于本文为回顾性研究，上述各组间人数差异较大，可能对统计结果有影响，因此结果尚需要前瞻性随机对照研究来进一步证实。

在Auperin等^[15]^进行的一项*meta*分析中证实PCI减少了而不仅是推迟了SCLC患者脑转移的发生。这一*meta*分析显示PCI使SCLC患者的3年生存率提高了5.4%。尽管这项*meta*分析中广泛期患者人数较少，但在局限期和广泛期患者中都看到了类似的结果。由EORTC组织的一项随机对照临床试验评估了286例对初始化疗有效的ES-SCLC接受PCI与未接受PCI的差异。与对照组相比，PCI组减少了脑转移的症状（14.6% *vs* 40.4%），提高了1年生存率（27.1% *vs* 13.3%）^[16]^。近期，有几项*meta*分析结果同样证实了PCI能够降低SCLC患者的死亡风险^[17, 18]^。目前SCLC NCCN指南中作为1类证据推荐：对于完全或部分反应的无论是局限期还是广泛期患者，都应进行PCI^[2]^。本研究结果显示PCI是保护性独立预后因素（RR=0.679, *P* < 0.01），能够降低32.1%死亡风险，显著延长ES-SCLC患者生存期（*P* < 0.01），与既往研究结果一致。但是本研究包含了脑转移和脑放疗患者，未行PCI组可能因初诊时脑转移，疾病分期更晚，本身具有更差的预后，对研究结果有干扰。

综上，化疗基础上的原发灶放疗、转移灶局部治疗和PCI能够降低ES-SCLC患者的死亡风险，延长生存时间。并且在转移灶迅速增长、出现症状前给予局部治疗更能使患者生存获益。原发灶放疗的时机、转移灶局部治疗对于ES-SCLC患者生存的改善，尚需要前瞻性Ⅲ期随机对照研究来证实。
